# Charting the transcriptomic landscape of primary and metastatic cancers in relation to their origin and target normal tissues

**DOI:** 10.1126/sciadv.adn0220

**Published:** 2024-12-06

**Authors:** Neel Sanghvi, Camilo Calvo-Alcañiz, Padma S. Rajagopal, Stefano Scalera, Valeria Canu, Sanju Sinha, Fiorella Schischlik, Kun Wang, Sanna Madan, Eldad Shulman, Antonios Papanicolau-Sengos, Giovanni Blandino, Eytan Ruppin, Nishanth Ulhas Nair

**Affiliations:** ^1^ Cancer Data Science Laboratory (CDSL), National Cancer Institute (NCI), National Institutes of Health (NIH), Bethesda, MD, USA.; ^2^ Fischell Department of Bioengineering, University of Maryland, College Park, MD, USA.; ^3^ Translational Oncology Research Unit, IRCSS Regina Elena National Cancer Institute, Via Elio Chianesi, Rome, Italy.; ^4^ Sanford Burnham Prebys Medical Discovery Institute, San Diego, CA, USA.; ^5^ Department of Comparative Biosciences, University of Illinois Urbana-Champaign, Urbana, IL, USA.; ^6^ Department of Bioengineering, University of Illinois Urbana-Champaign, Urbana, IL, USA.; ^7^ Laboratory of Pathology, National Cancer Institute (NCI), National Institutes of Health (NIH), Bethesda, MD, USA.

## Abstract

Metastasis is a leading cause of cancer-related deaths, yet understanding how metastatic tumors adapt from their origin to their target tissues remains a fundamental challenge. To address this, we assessed whether primary and metastatic tumors more closely resemble their tissues of origin or target tissues in terms of gene expression. We analyzed expression profiles from multiple cancer types and normal tissues, including single-cell and bulk RNA sequencing data from both paired and unpaired patient cohorts. Primary tumors were overall more transcriptomically similar to their tissues of origin, while metastases shifted toward their target tissues. However, pathway-level analysis highlighted critical metabolic and immune transcriptomic changes toward target tissues during metastasis in both primary and metastatic tumors. In addition, primary tumors exhibited higher activity in cancer hallmarks such as “Activating Invasion and Metastasis” when compared to metastases. This comprehensive analysis provides a transcriptome-wide view of the processes through which cancer tumors adapt to their metastatic environments before and after metastasis.

## Teaser

Both primary and metastatic tumors exhibit gene expression changes to better match target tissues in the metastatic process.

## INTRODUCTION

Most cancer deaths are caused by metastasis, a hallmark of cancer that remains poorly understood. The metastatic process, in which primary tumor cells adapt and travel from their origin tissue (OT) to target tissue(s) (TTs), is a complex cascade of molecular alterations (*1*). Numerous studies have examined the molecular features of metastatic cancers, highlighting hallmarks such as cell motility and invasion, the ability to modulate the TT, phenotype plasticity, and the ability to colonize the TT (*2*). In particular, metabolic adaptations play an important role in metastases as they seed in their TT (*3*), including altered fatty acid metabolism and changes in energy supply to initiate and promote metastasis (*4*–*7*). Primary tumors, on the other hand, have been found to remain overall metabolically similar in their expression profiles to their normal OTs, although with some up-regulation of pathways such as nucleotide synthesis and glycolysis (*8*), suggesting different genomic rewiring during growth from metastases.

Previous work has directly compared primary and metastatic tumors’ transcriptomics, identifying differentially expressed markers and metastatic alterations across various cancer types (*3*, *9*, *10*). Furthermore, genomic sequencing of 25,000 primary and metastatic tumors revealed higher chromosome instability, fractions of clonal mutations, and enrichments in drug resistance mechanisms in metastases compared to primary tumors (*11*). However, genomic or transcriptomic rewiring during metastasis is also dependent on the TTs in the metastatic process. Metastatic tumors are known for their particular organotropism, i.e., their specific spread to distant metastatic sites, with different cancer types displaying distinct distributions of TTs in metastasis (*10*). On this notion, specific genomic alterations were linked to the particular organotropisms in various cancer types, suggesting the importance of the TT in the metastatic process (*11*). Additionally, the OTs play a role as differential expression analysis of primary tumors from The Cancer Genome Atlas (TCGA) network and adjacent normal tissues exhibited heterogeneity of tricarboxylic acid cycle, anaplerotic reactions, and electron transport chain expression profiles, with unique expression changes specific to tumors from different OTs (*12*, *13*). Thus, both the OT and TT of a tumor influence growth and adaptations during metastasis.

There has been considerable work on this interplay of tumors and their corresponding normal tissues. Lee *et al.* (*14*) sequenced five sets of paired normal and primary/metastatic samples of patients with colorectal cancer (COAD) and liver metastases, finding cell cycle, mitosis, and cell division pathways to be enriched in both the primary carcinoma and metastases, with the tumors having similar expression overall. Comparison of single-cell transcriptome data of colorectal tumors (paired primary and metastatic) and paired normal OT and TT found PPAR peroxisome proliferator–activated receptor signaling pathway–related genes up-regulated in tumor epithelial cells compared to normal ones. Inhibiting this signaling pathway resulted in stunted colorectal tumor organoid growth in vitro, suggesting it as a tumor-specific target for COAD (*15*). Metastasis to specific target sites from various cancer types has also been studied through single-cell atlases, with the brain and liver being common sites, finding organ-specific markers of metastasis in normal tissues and contributing to the burgeoning understanding of tumor organotropism (*16*–*18*). Roshanzamir *et al.* (*19*) corroborate this notion, having used bulk transcriptomic data and metabolic network analyses of triple-negative breast cancer (TNBC) metastases to find that the metastases adapt their metabolism to various TTs while also retaining metabolic signatures associated with the primary TNBC. They found enrichment of metabolic pathways, e.g., bile acid metabolism, and immune response functions, e.g., coagulation, in metastatic tumors compared to primary tumors, while primary tumors were enriched more with proliferation, signaling, and inflammatory response pathways. Furthermore, their genome-scale metabolic model analysis of these TNBC metastases, their primary tumors, and normal (noncancerous) tissues highlighted the importance of several metabolic pathways like nicotinate and bile acid metabolism as potential pathways for targeting metastases.

With the growing interest in studying the molecular alterations occurring in cancer cells as they seed and adapt in their metastatic niche, we sought to identify pan-cancer patterns of transcriptomic similarities and differences to noncancerous OT and TT, which have not yet been characterized for primary and metastatic tumors in a genome-wide manner. Here, we conduct such a study, performing a large-scale, pan-cancer gene expression comparison between (i) a tumor’s OT, (ii) the primary tumor, (iii) the metastatic tumors, and (iv) their TTs. Studying this fundamental research question at this time is a nontrivial challenge as, to date, a large-scale, pan-cancer transcriptomic dataset containing per-patient fully paired sets of normal tissue, primary tumors, and metastatic tumors does not exist. Yet, we believe that it is timely to begin exploring this question as best as is feasible with the currently available data. To this end, we set out to conduct a few analyses in concert. We begin by comparing the relevant entities in a small quadruple-paired cohort (all four sample types from the same patient) and then analyze multiple paired (primary and metastatic only) cohorts and two large-scale unpaired cohorts of bulk transcriptomics, along with a paired single-cell transcriptomics dataset and computationally deconvolved bulk dataset. On the basis of these data, we aim to identify the underlying alterations that drive metastasis across cancer types in a robust, consistent manner as possible. A summary of all datasets used in this work is provided in table S1A.

Given these datasets, we asked three research questions: (i) Are primary and metastatic tumors transcriptionally more similar to the organ from which they originate (termed the OT) or to the organ to which they metastasize (termed the TT)? (ii) On the pathway level, which pathways’ expression in metastatic tumors is most similar to their TT for a given primary cancer type? (iii) Are primary cancers primed for metastasis before leaving the tissue of origin? This knowledge can provide a genome-wide view on how cancer cells adapt to their target organ microenvironment; pathways closer to the TTs in metastatic tumors but not in primary ones point to a metastatic niche adaptation, while pathways closer to the OTs in metastatic tumors suggest conserved oncogenic transcriptomic signatures.

## RESULTS

### Overview of the analysis

**Fig. 1.**
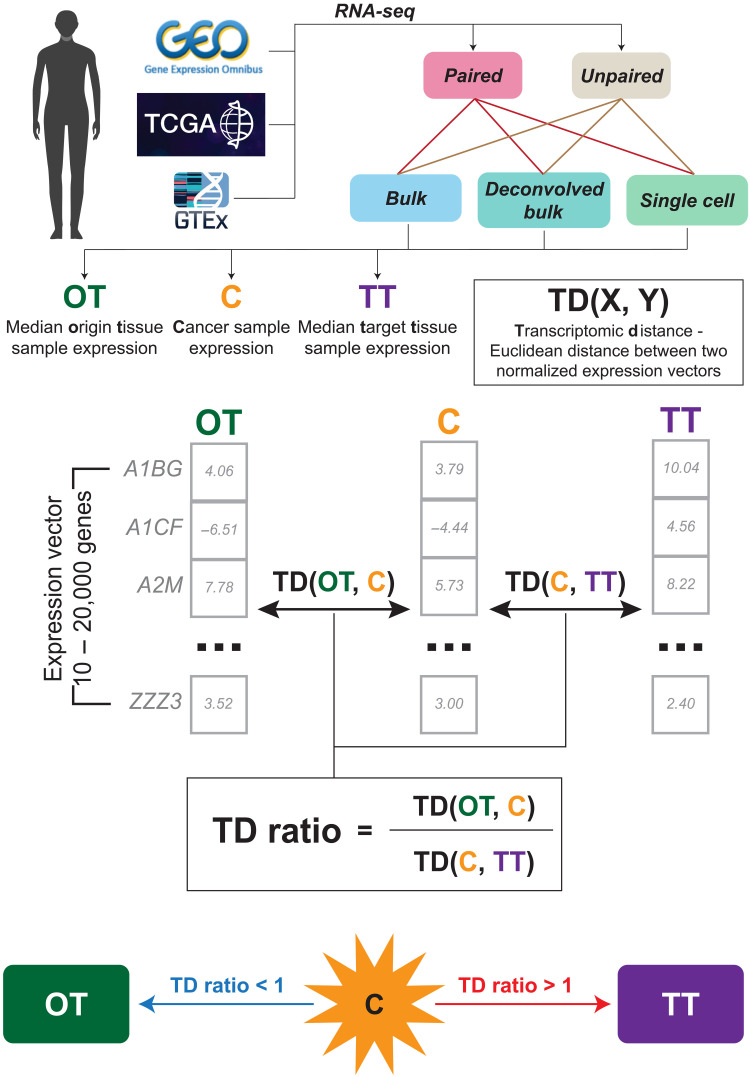
An overview of the approach, depicting the four entities studied in our pipeline. Healthy tissue from the primary tumor site, termed OT, primary tumors that have been clinically annotated as metastasizing to a particular target organ, metastatic samples, and healthy TT to which the tumor metastasizes. Data were sourced from several sources and are summarized in table S1A. The TD(X, Y) of a given tumor sample X denotes the distance of its expression from the median expression of a given origin X and TTs Y. Last, the transcriptomic distance ratio (TD ratio) denotes whether a cancer sample is more similar to the TT (>1), or to the OT (<1). These TD ratios are used in the downstream analysis to compare primary and metastatic samples on the whole-genome and pathway levels.

To study whether the overall gene expression of primary and metastatic tumors is closer to their tissue of origin or to their TT, we acquired datasets from publicly available sources such as the Gene Expression Omnibus (GEO), the Cancer Genome Atlas (TCGA) (*12*), and the Genotype-Tissue Expression (GTEx) (*20*) atlas, sourcing the gene expressions for four key entities: (a) primary tumors either annotated as having metastases or having paired metastases from the same patient, (b) metastatic tumors, (c) normal, noncancerous tissues of origin (OTs), and (d) normal, noncancerous TTs (table S1A). Except for the one quadruple-paired bulk RNA sequencing (RNA-seq) dataset and the paired single-cell RNA-seq dataset, all noncancerous OT and TT used in analyzing cohorts were sourced from the GTEx. To ensure comparability, each set of analyzed gene expression datasets was normalized in a uniform fashion (Materials and Methods). We then compared the expression of tumor samples to the expression of the corresponding OT and TT. Similarity was quantified by computing the Euclidean distance between the tumor and normal expression profiles of the primary or metastatic tumors to the median expression of their corresponding normal tissue of origin and TTs—termed their transcriptomic distance (TD) (Materials and Methods). For each tumor sample’s expression profile (either primary tumor or metastasis), we calculated its “TD ratio” by computing the ratio of its TD to the tissue of origin over its TD to the TT*.* Euclidean distance was used as a measure to compute TD, but we also tested the robustness of our approach by using alternative measures like Spearman’s correlation and cosine similarity–based distance to validate our approach (Materials and Methods). Furthermore, we computed the TD ratios for 50 key cellular pathways from MSigDB’s hallmark gene sets (*21*) in each sample, quantifying which pathways are closer to the OT or to the TT than expected by chance, as well as the expression activity of the 10 main cancer hallmarks (*22*) in primary and metastatic samples (Materials and Methods). Figure 1 provides a visual overview of the TD ratio calculation.

### The landscape of primary and metastatic tumor TD ratios in paired cohorts

To test whether paired primary and metastatic tumors are transcriptionally more similar to their OT or TT, we begin by examining the TD ratios of primary tumors and their paired metastases (present along with the primary or on patient follow-up; details in table S1F) from several bulk-paired RNA-seq expression cohorts—all summarized in table S1A. Given the organotropic nature of cancer metastases (*3*, *10*), we organized our analyses by TT, starting with liver metastases.

#### Comparison of paired primary and metastatic tumors expression to OTs and TTs using bulk RNA-seq cohorts

**Fig. 2.**
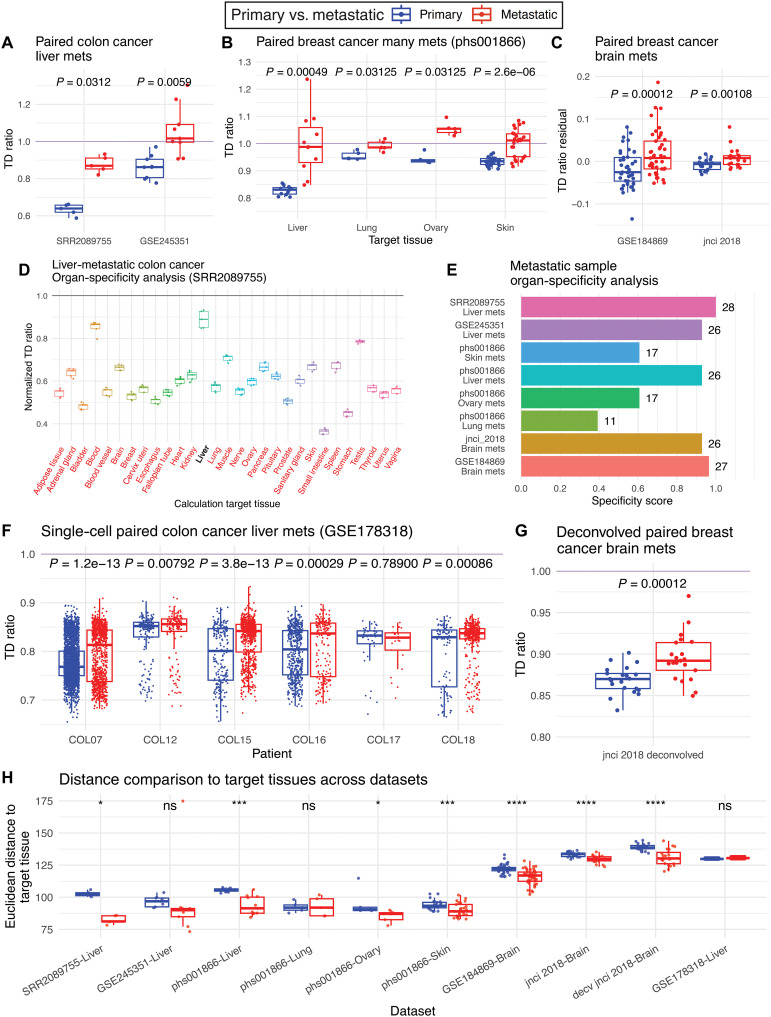
Transcriptomic landscape of primary and metastatic tumors in relation to their corresponding noncancerous tissues across bulk RNA-seq, paired cohorts. All datasets are summarized in table S1A. In general, metrics corresponding to primary samples are in blue, red for metastases (“Mets”). (**A** to **C**) Landscape of TD ratios for the colon cancer liver metastases, BRCA with multiple-site metastases, and BRCA brain metastases datasets, respectively. (**D**) Normalized TD ratios calculated using colon cancer liver metastases samples and various TTs (TT) sourced from GTEx. The true liver TT is bolded, while any other TT marked red has normalized TD ratios significantly lower than the true liver TT. (**E**) Bar plot quantifying what is found in (D) and expanding the specificity analysis (Materials and Methods) to all of the bulk paired datasets, showing specificity score and the number of TTs with lower normalized TD ratios compared to the true TT at the end of each bar. (**F** and **G**) The same as (A) to (C) but for epithelial cells of single-cell colon cancer liver metastases (stratified by patient) and deconvolved BRCA brain metastases datasets. (**H**) Landscape of Euclidean distances for primary and metastatic tumors in each dataset. All *P* values were generated by the one-sided Wilcoxon signed-rank test for these paired datasets, testing whether metastatic sample metrics were greater than primary ones [but opposite direction for the Euclidean distances in (H)]. Significance levels: *P < 0.05, ***P < 0.001, ****P < 0.0001. ns, not significant.

We first analyzed a dataset of five quadruple-paired COAD samples, that is, five primary colon, five metastatic liver, and five corresponding OT (colon) and TT (liver) samples (termed SRR2089755) (*14*). While the bulk expression of both primary and metastatic cancers is closer to that of the OT (TD ratios <1), the metastatic cancer expression is significantly closer to that of the TT than the primary tumor expression (one-sided Wilcoxon signed-rank test, *P* = 0.031; Fig. 2A). This same trend (one-sided Wilcoxon signed-rank test, *P* = 0.0059) is found in another paired colon cancer cohort of nine samples (termed GSE245351) (*23*), where metastases, unlike their paired primaries, exhibited TD ratios closer to and around 1 (Fig. 2A). These results testify to an expression shift to adapt to the target liver tissue, while the primary cancer’s expression remains more like the origin colon tissue’s expression.

An analogous analysis of 46 paired primary and metastatic samples from patients with breast cancer (BRCA) (termed phs001866) (*24*) demonstrated similar findings. These patients with BRCA had metastases to the liver (*n* = 22), lung (*n* = 10), ovary (*n* = 10), and skin (*n* = 50). Using the bulk expression of normal breast tissue (OT) and normal liver, lung, ovary, and skin tissues (TTs) from the GTEx, TD ratios were calculated for each primary sample and its paired metastasis (Materials and Methods). Across all four TT sites, the metastatic tumors were closer to their TT in expression than OT when compared to the primary tumors (one-sided Wilcoxon signed-rank test, false discovery rate (FDR) < 0.1; Fig. 2B). The ovary metastases exhibited TD ratios all above 1, indicating an overall higher closeness in expression to the ovary TT than the breast OT. The liver, lung, and skin metastases, which all exhibited TD ratios around 1, suggest a “midway” transcriptomic state between the OT and TT.

As our analysis is based on bulk RNA-seq data containing expression from a variety of cell types, we tested whether our results are confounded by differences in the tumor’s malignant cell fraction, also known as its “tumor purity.” Reassuringly, we did not find a meaningful correlation between the estimated tumor purity and TD ratio for the datasets analyzed so far (Materials and Methods and table S1D). However, when analyzing two other paired BRCA brain metastatic cohorts (*n* = 45, termed GSE184869, and *n* = 21, termed jnci 2018) (*25*, *26*) and calculating the samples’ TD ratios, significant correlations were found between the TD ratios and tumor purity scores (*P* < 0.05; table S1D). Since tumor purity was a possible confounding factor in the analysis of these cohorts, we explicitly regressed out the tumor purity component from the TD ratio via a linear model and acquired the “TD ratio residuals,” which represented the TD ratio values free from tumor purity confounding effects (Materials and Methods). Despite the need for tumor purity regression, both the GSE184869 and jnci 2018 datasets exhibited the same trends as the cohorts reported above, in that the metastatic TD ratio residuals were higher than the primary samples’ TD ratio residuals (*P* < 0.05; Fig. 2C). Moreover, we also repeated our analysis using non-Euclidean distance measures (for computing TD ratio) such as Spearman’s correlation–based and cosine similarity–based measures, which are more immune to any batch effects between datasets, to obtain similar results, thus showing the robustness of our approach (fig. S1 and notes S1).

Given the organotropic nature of cancer metastases (*3*, *10*, *18*), we hypothesized that the metastatic samples may show transcriptomic adaptations specific toward their TT as opposed to some other noncancerous tissue. To check whether metastatic tumors have specific transcriptomic shifts to their true TTs, we computed a normalized TD ratio for each metastatic tumor that accounts for TD between the tissue of origin and TT expression along with the distance between the tumor and TT expression (“Specificity analysis,” details in Materials and Methods).

Starting with the COAD liver metastases dataset (SRR2089755), we performed this specificity analysis in which we calculated normalized TD ratios for each sample fixing the OT to colon and varying the TT (Materials and Methods). Supporting our hypothesis, we find that the liver metastases’ normalized TD ratios are higher when using the actual target normal liver tissue as the TT compared to using any of the other 28 other normal tissues present in the GTEx as the TT, thus suggesting a specific transcriptomic alteration toward their true TTs (one-sided Wilcoxon signed-rank test, FDR < 0.1; Fig. 2D). Extending this to all bulk-paired datasets analyzed so far, we defined a score for each dataset indicating for how many TTs the normalized TD ratios were greater when using the true TT versus the other possible TTs (Fig. 2D and Materials and Methods). We found mixed results, where liver metastases from the colon along with liver and brain metastases from the breast have higher specificity in their transcriptomic alterations toward their respective TTs, whereas BRCA skin, lung, and ovary metastases from the same cohort show lower specificity (Fig. 2E).

#### Reinforcing our findings on the paired bulk RNA-seq analysis using a paired single-cell and deconvolved bulk RNA-seq cohorts

Single-cell RNA-seq provides an opportunity to examine the transcriptomic adaptions of primary tumors and their metastases on the cell-type level, allowing us to perform our analysis in a tumor purity–free manner and home in on the alterations found in just the tumor cells of cancer samples. To this end, we analyzed a paired COAD single-cell cohort with liver metastases (*n* = 6 patients, ~6500 cells, termed GSE178318) (*27*). Normal OT (colon) and TT (liver) comparators were taken from Lee *et al.* (*28*) and the Tabula Sapiens database (*29*), respectively. A TD ratio was calculated for each cancer epithelial cell in GSE178318 using 10,000 most variable genes (Materials and Methods and fig. S2). For five of the six patients, the metastatic cells expressed more similarly to the TT than primary cells (FDR < 0.1), suggesting transcriptomic alterations toward the TT in the metastatic cells (Fig. 2F). Reassuringly, when compared to the quadruple-paired bulk COAD liver metastases dataset, both cohorts show that metastatic samples have greater TD ratios than primary samples (Fig. 2A).

Recent computational expression deconvolution methods have also allowed for the imputation of cell type–specific gene expression from a set of bulk tumors. This process provides another way to analyze the transcriptomic alteration found in tumor cells specifically. The paired BRCA brain metastases dataset jnci 2018, analyzed previously in Fig. 2C, was deconvolved into cell type–specific gene expression using both a signature for primary BRCA samples and metastatic BRCA brain metastases via the “COnfident DEconvolution For All Cell Subsets” (CODEFACS) algorithm (Materials and Methods) (*30*). After determining the gene expression specific to the cancer epithelial cells in the cohort, a TD ratio was generated for each sample. Reassuringly, this dataset that was once confounded by tumor purity concerns no longer had significant correlations between sample tumor purity and TD ratio after deconvolution (table S1D). As seen in Fig. 2G, the expression of brain metastases is significantly closer to the brain TT’s expression than the expression of primary breast samples, but both primary and metastatic samples are overall closer in expression to the breast OT (FDR < 0.1).

Across all datasets, we see a consistent trend of higher TD ratios in metastatic samples than in the paired primaries. Although the specificity of such adaptation appears to vary depending on the cancer type studied, the overall transcriptomic shift toward to the TT in metastases compared to primary tumors remains consistent. This notion is further supported by finding lower Euclidean distances between the metastatic samples and their TTs than those observed for the primary samples in most of the paired datasets (Fig. 2H). Reassuringly, across these datasets, dimensional reduction of the gene expression profiles of samples does not result in clusters separated by primary and metastatic classification (for each cancer type), suggesting that these results are due to biological difference and not due to batch effects (fig. S3).

### The overall landscape of primary and metastatic tumor TD ratios

Given the typically small and limited size of paired datasets, we further explored large-scale, unpaired, pan-cancer bulk RNA-seq cohorts to assess transcriptomic alterations across a broader spectrum of cancer types while acknowledging the inherent limitations of this unpaired data. This analysis included: (a) primary tumors of 10 different cancer types sequenced from patient samples that have been annotated as metastasizing to various TTs (collected from the TCGA, *n* = 306); (b) metastatic tumors of 14 different cancer types biopsied from five main TTs (collected from the MET500 collection, *n* = 194); and (c) normal, noncancerous tissue samples, composing both the OT and TT (GTEx, *n* = 5663) (*12*, *20*, *31*). Only samples where metastasis is known to have occurred, either at the time of biopsy or later on, were considered from the TCGA. To ensure comparability between the three RNA-seq datasets, they were normalized similarly such that gene vectors were in the same format during TD ratio calculation (Materials and Methods).

**Fig. 3.**
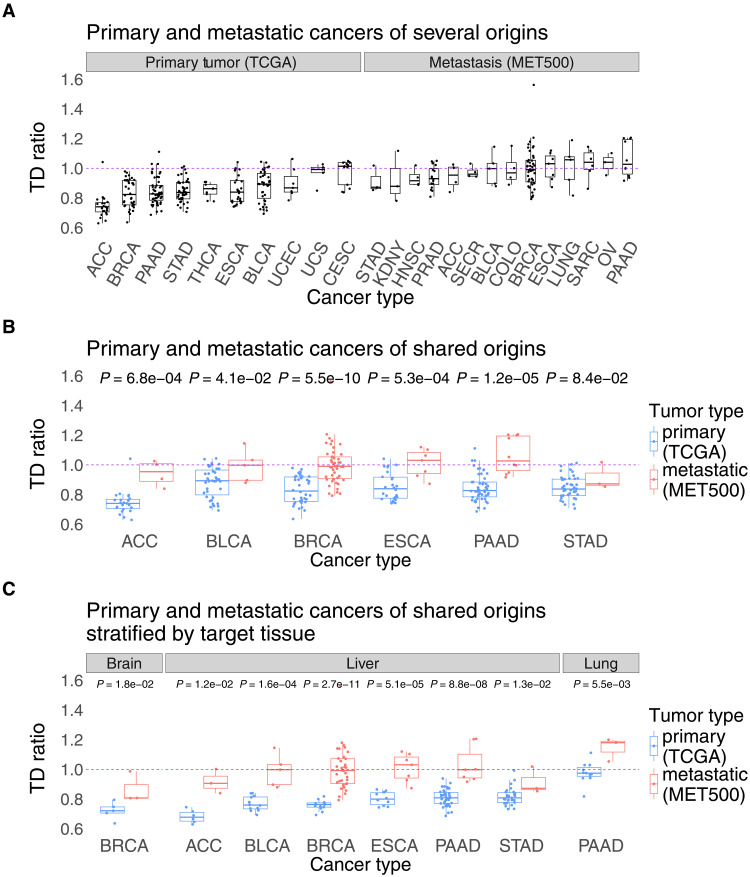
Transcriptomic landscape of primary and metastatic tumors compared to their corresponding noncancerous tissues across bulk RNA-seq, unpaired cohorts. Tumor samples (primary from TCGA, metastatic from MET500) with TD ratios above 1.0 are closer to the TT, while those with TD ratios below 1.0 are closer to the OT; the purple line marks the equipoise value of 1.0 (*12*, *31*). In (**A**), the TD ratios (*y* axis) are plotted for all primary and metastatic samples of a given cancer type and ordered by TD ratio. For the same TD ratios but stratified by TT of metastasis, see fig. S5. In (**B**), all samples are plotted for cancer types with at least three samples in both datasets (for any TT). (**C**) is the same as (B) except the samples are stratified by TT. The cancer types in the *x* axis are mentioned as TCGA or MET500 abbreviations, and their full forms are provided in table S1E (e.g., COAD, colorectal cancer). All *P* values presented are the result of one-sided Wilcoxon rank-sum tests for these unpaired datasets. FDR value <0.1 in all cases presented in (B) and (C).

As before, we calculated and examined the TD ratios of primary and metastatic tumors, the results of which are summarized in Fig. 3. Figure 3A shows TD ratios of the primary and metastatic tumors across several cancer types. Consistent with the paired analysis, the transcriptomes of most primary tumors are closer to that of their corresponding tissue of origin than to that of their eventual metastatic targets, except for primary uterine carcinosarcoma and cervical squamous cell carcinoma that have a more equipoise position between their OT and TT (TD ratio ~ 1). The transcriptomes of metastatic tumors generally have an equipoise position, with metastases originating from stomach cancer representing the closest to origin (median TD ratio = 0.82) and those from pancreatic cancer representing the furthest from origin (median TD ratio = 1.03).

In all six cancer types for which we had sufficient data for primary and metastatic tumors of the same cancer type, the metastatic tumors are closer in expression to the TT than the primary tumors that have been reported to have eventually metastasized to that TT (FDR < 0.1; Fig. 3B and Materials and Methods). This same trend is found when considering only primary samples metastasizing to the brain, liver, or lung (we had most samples for these three TTs) versus metastases to those sites. The brain and lung metastases subsets only had two primary cancer types with sufficient numbers of samples to make a comparison (breast and pancreatic cancer, respectively), while the liver metastases subset had enough samples from six primary cancer types. In all cases, the metastatic tumors adhere to the aforementioned trend compared to the primary tumors (FDR < 0.1; Fig. 3C). These results are not confounded by tumor purity (Materials and Methods and table S1D). Furthermore, the key findings remained similar when replacing the Euclidean distance method for calculating TD ratios with Spearman’s correlation–based and cosine similarity–based distance measures (notes S1 and fig. S4).

### Pathways analysis and cancer hallmark activities for different cancer types and TTs

We next performed a higher-resolution analysis to compute the TD ratios at the pathway level to identify key biological pathways in primary tumors and metastases whose gene expression either is more like that of the TT or remains similar to the OT. Using these ratios, we were able to compute the median pathway–specific TD ratios separately across primary and metastatic samples for each cancer type. Empirical *P* values (and FDR-adjusted values) for each of these TD ratios were computed using random genes with the same size of that pathway. Furthermore, we introduced a comparative metric, the Δ pathway–specific TD ratio, to evaluate the extent by which the pathway-specific TD ratio diverges from the TD ratio computed using all genes (Materials and Methods). A Δ pathway–specific TD > 1 implies that the median pathway–specific TD ratio for all samples is greater than expected in comparison to the all-gene analysis (i.e., the transcriptome of the pathway genes of the tumor is more similar to that of the TT than the OT in comparison to the all-gene analysis). A Δ pathway–specific TD < 1 implies the opposite: The transcriptome of the pathway genes of the tumor is more similar to that of the OT than the TT in comparison to the all-gene analysis (fig. S6). Of particular interest were pathways of two main types: (a) those whose Δ pathway–specific TD ratios that flipped from low (<1) to high (>1) (or vice versa) between the primary and metastatic groups, marking a significant shift in the pathway’s expression during metastasis, and (b) those that have high Δ pathway–specific TD ratio in primary cancers, potentially marking ways the primary cancer is preparing for metastasis via expression changes from its OT.

#### Pathway-specific alterations point to key adaptive transcriptomic changes in metastases

**Fig. 4.**
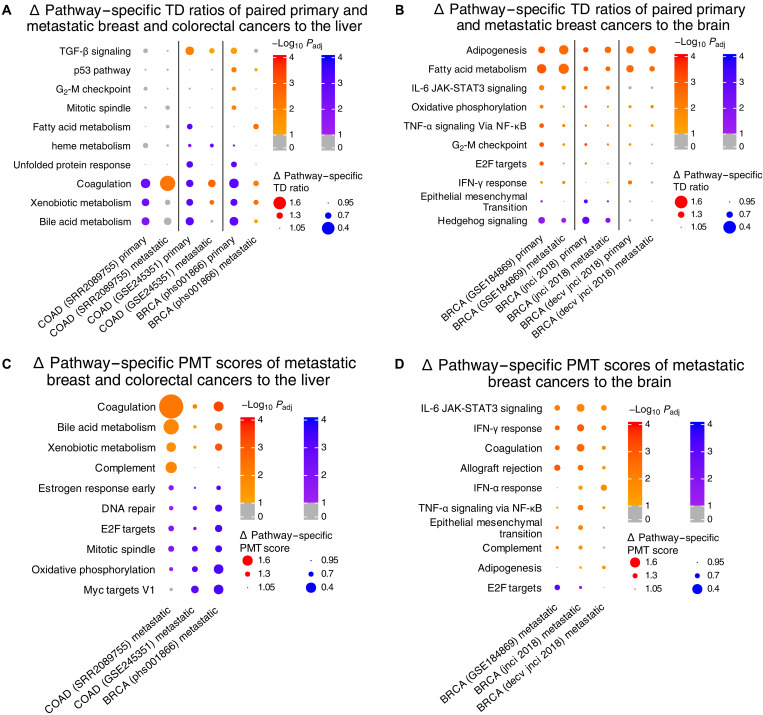
Pathway expression similarity in paired primary and metastatic tumors. All datasets are summarized in table S1A. (**A** and **B**) Heatmap of (A) liver metastatic and (B) brain metastatic cohorts with *x* axis labeled with dataset and cancer type, and hallmark pathways on the *y* axis, where each dot is sized by Δ pathway–specific TD ratio’s distance from the equipoise value of 1. (**C** and **D**) Heatmaps of the same cohorts from (A) and (B), respectively, but now using PMT score (Materials and Methods). Red color is used for Δ pathway–specific TD ratios/PMT scores above 1 (closer to TT, normalizing for the score of the whole transcriptome), while blue color is used for those below 1 (closer to paired primary tumor, normalizing for the score of the whole transcriptome). In all four subfigures, the intensity of the color is determined by significance after empirical *P* value calculation and FDR correction (Materials and Methods). More richly colored dots imply that a metric value (TD ratio/PMT score) is further from 1 than expected by chance, i.e., more significant (FDR < 0.1), with insignificant metric values colored in gray. Not all pathways are shown here; pathways were prioritized on the basis of significance across cancer types for plotting. All Δ pathway–specific TD ratio and Δ pathway–specific PMT score data pertaining to this analysis can be found in table S2 (A to D) (one table per subfigure).

First, we analyzed liver metastases from the paired COAD and BRCA datasets. The coagulation pathway demonstrates this flipped Δ pathway–specific TD ratio behavior, having a low Δ pathway–specific TD ratio in COAD and BRCA primary samples, but then having a high Δ pathway–specific TD ratio in most of the liver metastases, reflecting expression changes to better match the liver TT in the metastatic state (FDR < 0.1; Fig. 4A and table S2A). This same finding is extended in the large-scale MET500 dataset, where both coagulation and bile acid metabolism pathways in BRCA liver metastases have high Δ pathway–specific TD ratios (FDR < 0.1; fig. S7A and table S4A). These findings are in agreement with previous work in BRCA highlighting changes in bile acid metabolism and coagulation to support metastasis (*19*), with the activation of the coagulation cascade also being known to play an important role in the metastatic process in many cancer types (*32*), overall reaffirming the notion that these pathway alterations are important in metastasis. Liver metastases from lung cancers, esophageal adenocarcinomas, and adrenocortical carcinomas in the MET500 show similar results for these two pathways, suggesting their importance in cancerous origins beyond the breast and colon (FDR < 0.1; fig. S7A and table S4A). Of further interest in the breast (dataset phs001866), the adipogenesis and fatty acid metabolism pathways show a similar high ratio pattern in subsets of skin, liver, ovary, and lung metastases, suggesting the use of these pathways in adapting to the various TTs (FDR < 0.1; fig. S7B and table S4B). Lipid metabolism, a metabolic process that involves these two particular pathways, has indeed been shown to be important in the metastatic process for multiple cancer types (*33*). While the BRCA brain metastases also exhibited lipid metabolism pathway expression closer to the brain TT, immune pathways such as interleukin-6 (IL-6) Janus kinase–signal transducers and activators of transcription 3 (JAK-STAT3) signaling and tumor necrosis factor–α (TNF-α) signaling via nuclear factor κB (NF-κB) followed the same trend (FDR < 0.1; Fig. 4B, table S2B, fig. S7C, and table S4C). Both TNF-α and IL-6 signaling have been shown to play protumorigenic and prometastatic roles in BRCA, particularly with secretion from/to cancer cells (*34*, *35*). These metastases appear to use these pathways to better adapt to the environments of their TTs.

To track these alterations more closely, we built a score to compare their similarities to their paired primary tumors and TTs. This primary metastatic target (PMT) score denotes the ratio of Euclidean distances between the gene expression vectors of the metastatic samples and their primary tumors over the same distances computed versus their TTs (Materials and Methods). At the whole transcriptome level, we see expectedly that the metastatic samples are overall closer to their paired primary tumors than the normal noncancerous TTs (fig. S8). To chart these alterations on a pathway level, we computed a Δ pathway–specific PMT score, which was derived in a similar fashion as the Δ pathway–specific TD ratio described above. A Δ pathway–specific PMT score > 1 implies that the pathway is closer in expression to the TT than the paired primary tumor, normalizing for the shift in the entire transcriptome (Materials and Methods). Meanwhile, a Δ pathway–specific PMT score < 1 implies the opposite. We were primarily interested in the pathways with high Δ pathway–specific PMT scores (above 1), i.e., those becoming more similar to the TT.

Reassuringly, we found good agreement with the Δ pathway–specific TD ratio analysis above. Once again in the paired COAD and BRCA liver metastases, coagulation and bile acid metabolism expressed more similarly to the TT even when compared to their paired primary tumors (FDR < 0.1; Fig. 4C and table S2C). The same was true for the adipogenesis, IL-6 JAK-STAT3 signaling, and TNF-α signaling via NF-κB pathways in the BRCA brain metastases (FDR < 0.1; Fig. 4D and table S2D)—all highlighted as closer to the TT in the Δ pathway–specific TD ratio analysis of the same data. Overall, these are pathways that are being altered toward the TT compared to both paired primary tumors and the OT alike, suggesting ways the metastases are adapting to and inheriting advantages at the TT site.

#### Across cancer types, primary tumors already show gene expression that better matches TTs of metastasis

We observed that the expression of fatty acid metabolism and adipogenesis pathways is also of interest in primary cancers. In BRCA primary tumors with both paired and later-developed brain metastases, these two metabolic pathways have high Δ pathway–specific TD ratio, indicating more closeness in expression to the brain TT than the breast OT. This suggests that these lipid metabolism pathways are critical in all stages of the BRCA brain metastasis process, that is, before and after metastasis (FDR < 0.1; Fig. 4B, table S2B, fig. S7C, and table S4C). Consistent with our findings, lipid metabolism is known to be necessary for proper formation of brain metastases, and metastasis-initiating cells (MICs) in BRCA have been shown to be dependent on fatty acid usage in particular for growth (*3*, *36*). Oxidative phosphorylation (OXPHOS) pathway has been strongly correlated with brain metastasis risk in patients with lung cancer (*37*). Aligned with that, we find that OXPHOS shows greater similarity in expression to the brain TT than the breast OT in our deconvolved BRCA brain metastases (FDR < 0.1; Fig. 4B). This also corroborates with the notion that breast MICs, shown to preferentially use OXPHOS over glycolysis, rely on this pathway for metastatic progression (*36*). In addition, very recently, Terekhanova *et al.* (*38*) linked TP53 and TNF-signaling pathways to cancer initiation. Our primary BRCA analysis identifies these pathways closer in expression to the liver (p53 pathway) and brain (TNF-α signaling via NF-κB) TTs (FDR < 0.1; Fig. 4, A and B). These primary tumors appear to be adapting to the TT before they even reach it.

**Fig. 5.**
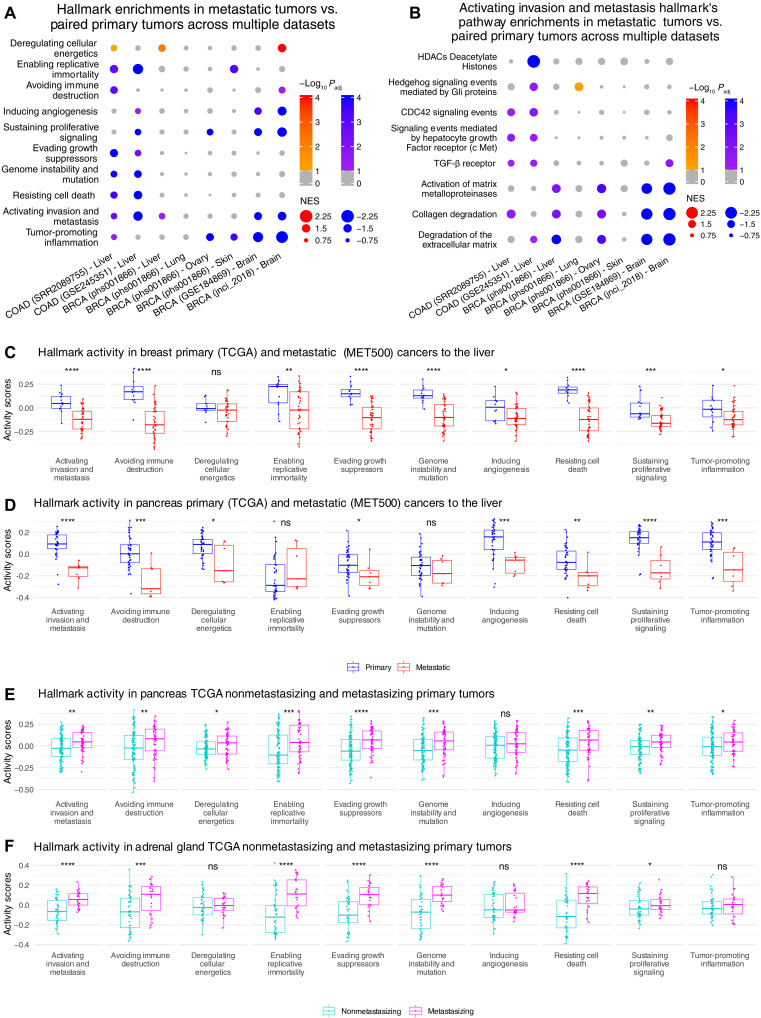
Cancer hallmarks’ activities in primary and metastatic tumors. (**A**) Heatmap of pathway enrichments from GSEA run on various datasets (delineated on the *x* axis using the format “cancer type (dataset) – target tissue”) and for various pathways shown on the *y* axis. The dots are sized by the GSEA NES, and colored red if the metastatic samples were enriched (NES > 0) or blue if the primary samples were enriched (NES < 0). More richly colored (more red/blue) dot imply a more significant enrichment (FDR < 0.1 as threshold). (**B**) The same as (A) but using pathways showing enrichment that compose the “Activating Invasion and Metastasis” hallmark. All NES and FDR values pertaining to (A) and (B) can be found in table S3 (A and B), respectively. (**C** and **D**) GSVA activity scores for the same cancer hallmarks for primary (blue) and metastatic (red) breast (C) and pancreatic (D) cancer, respectively. (**E** and **F**) GSVA activity scores for the same cancer hallmarks for nonmetastasizing (cyan) and metastasizing primary pancreatic (E) and adrenocortical (F) cancer, respectively. The significance levels used in (C) to (F) are *P < 0.05, **P < 0.01, ***P < 0.001, ****P < 0.0001.

In addition to the hallmark pathways from MSigDB, we considered the changes between paired primary and metastatic samples for the 10 principal hallmarks of cancer proposed by Hanahan and Weinberg (*22*). Here, we analyzed hallmark expression activity between primary and metastatic tumors instead of pathway similarity to normal tissues, and to determine whether each hallmark was more active (enriched) in primary or metastatic samples, we applied gene set enrichment analysis (GSEA) (*39*) on each dataset for gene lists composing those hallmarks (Materials and Methods) (*40*). Colon cancer samples saw primary tumor enrichment in 8 (dataset SRR2089755) and 7 (dataset GSE245351) of 10 hallmarks, while the two BRCA brain metastatic datasets saw primary tumor enrichment in 4 of 10 shared hallmarks. The BRCA dataset with four TTs saw the least significant enrichments, averaging only two enriched hallmarks per TT [normalized enrichment score (NES) < 0, FDR < 0.1; Fig. 5A and table S3A]. Unexpectedly, nearly all of these cancer hallmarks, when significantly enriched, are more active in the primary samples than their paired metastases. Only the deregulating cellular energetics hallmark is enriched in three of the metastatic cohorts (NES > 0, FDR < 0.1; Fig. 5A and table S3A). These hallmarks of cancer delineate critical functions of cancer cells agnostic of cancer type or TT, yet we find their overall activities to be lower in metastases, suggesting a decrease in use after metastasis. When further investigating the activating invasion and metastasis hallmark by running the same GSEA on the 28 individual pathways composing it, we found once again that the pathways, particularly those supporting cell motility and extravasation into the blood, were mainly enriched in primary samples (Fig. 5B and table S3B). This unexpected result is not due to some inherent bias in the data as no selective enrichment was found toward primary or metastatic samples when using random gene sets (fig. S9A and notes S2). Thus, these findings indicate that primary samples, as suggested by their higher levels of activating invasion and metastasis activity, are being primed for the metastatic process, after which such processes are less used.

Extending this analysis to the large-scale primary (TCGA) and metastatic (MET500) sample cohorts, we performed gene set variation analysis (GSVA) (*41*) of the cancer hallmarks on liver metastatic samples and primary samples from cancer types shared between the two cohorts to examine activity differences. As with the paired cohorts, most of the hallmarks showed higher activity scores in primary tumors, particularly for activating invasion and metastasis, than in metastatic tumors of the same cancer type and TT (one-sided Wilcoxon rank-sum test *P* < 0.05; Fig. 5, C and D; details provided in notes S3 and fig. S9, B to E). Within the TCGA, we also compared the hallmark activities of these primary tumor samples with marked metastases to those primary tumors from the same cancer type that do not have marked metastases. Reassuringly, we found higher trends of the activation invasion and metastasis hallmark’s activity in primary samples with marked metastases (table S3C). In the cancer types (pancreatic and adrenocortical) with the least imbalance of nonmetastasizing and metastasizing primary samples, we found significantly higher activity scores for multiple hallmarks in the samples with marked metastases (one-sided Wilcoxon rank-sum test *P* < 0.05; Fig. 5, E and F). Overall, we find that metastatic samples, although known to retain features of their OTs (*13*, *19*, *33*), unexpectedly show lower hallmark activities after they leave their tissue of origin.

## DISCUSSION

This study performs a large-scale, pan-cancer transcriptomic landscape analysis comparing primary and metastatic tumors to their normal origin and target tissues in the metastatic process. Although prior studies have attempted such comparisons, they have been limited to one cancer type or do not compare the cancers with both relevant noncancerous tissues. We first studied the transcriptomic alterations in a small but quadruple-paired cohort of bulk RNA-seq samples, representing the most clinically relevant case. From there, we analyzed several paired primary and metastatic cohorts, and, then, given the paucity of paired, multicancer datasets available, we used several of the largest publicly available datasets to define aggregate datasets to study expression similarity at the level of cancer type and tissue, integrating bulk, single-cell, and bulk deconvolved data types. Last, we examined transcriptomic alterations on the pathway and cancer hallmark level, dissecting changes in both primary and metastatic tumors.

Overall, we find that metastatic tumors across cancer types generally have an equipoise transcriptomic similarity between their tissue of origin and their metastatic TT and significantly greater similarity to the TT than the similarity of the corresponding primary tumors of the same type. In contrast, we found that primary tumors, overall, are largely transcriptionally similar to their tissue of origin, which concurs with the current notion that primary tumors remain functionally akin to their tissues of origin in many cancer types (*8*, *19*). The transcriptomic shift in metastases may also be specific to the TT of metastasis as shown in the colon cancer liver metastases and BRCA liver and brain metastases datasets—a result in line with the organotropic nature of metastasis (*3*, *10*). Strikingly, despite the different TTs and datasets analyzed across the bulk, deconvolved bulk, and single-cell levels, the trend of adapted metastases and more conserved primary tumors remained strong at this whole-transcriptome level.

When comparing the expression profiles of pathways in primary and metastatic tumors to their normal OT and TT, two main trends emerged. (i) Metastases adapt to better match their TTs. Colon and BRCA liver metastases tend to have bile acid metabolism and coagulation pathway expression more akin to that of the liver than that of both their respective OTs and paired primary tumors. The same is true for lipid metabolism pathway expression (e.g., adipogenesis) in BRCA brain, lung, ovary, and skin metastases, with a particular occurrence of immune pathways (e.g., IL-6 JAK-STAT3 signaling and TNF-α signaling via NF-κB) in the brain metastases. Such adaptations of functional pathways to better match the TTs’ environments coincides with findings current metastasis literature (*3*, *10*, *19*, *33*). (ii) Primary cancers are primed for metastasis, although they remain largely similar in expression to the OT. Along with the same adipogenesis and fatty acid metabolism pathways, OXPHOS and TNF-α signaling via NF-κB express more similarly to the brain than the breast in primary breast samples, highlighting perhaps preparatory mechanisms for metastasis. Comparisons of primary and metastatic tumors directly have pointed to the supportive role of these pathways in the metastatic process (*19*, *36*–*38*). Furthermore, notably, primary tumors are enriched for several cancer hallmarks, e.g., activating invasion and metastasis, when compared to metastatic tumors of the same cancer type. Overall, we see that tumors may adapt to metastasis both before and after the process occurs, and accounting for these two trends along with the highlighted pathways may help in the effort to impede metastasis formation.

Our study has a few limitations, some which we have addressed. Tumor purity is a potential confounding factor for the comparative transcriptomic analysis in the bulk as some surrounding normal tissues could be a part of the cancer biopsy. Therefore, we carefully checked and controlled for any meaningful associations between tumor purity estimates and TD ratios for both the unpaired and paired datasets. Furthermore, we reinforced the trends presented via corroboration of TD ratios calculated with single-cell and deconvolved bulk RNA-seq—both free of tumor purity issues. Notably, however, measures such as TD ratios or Euclidean distances are based on the entire transcriptome and do not capture gene-specific differences between primary/metastatic tumors and their OTs/TTs, hence, the motivation to create a TD ratio at the pathway-level to capture more functional differences and similarities to the OTs/TTs. Moreover, this pathway-specific analysis identifies pathways that show alterations over the whole transcriptome–based analysis, leaving it unaffected by tumor purity concerns.

Because of the lack of a large-scale transcriptomic dataset containing paired sets across several cancer types, we carried out a significant fraction of the analysis using aggregate, nonpaired datasets. One such dataset was TCGA, and while a very small subset of the TCGA primary samples were annotated as metastasizing to multiple TTs, we were unable to study them deeper because of limited sample size. Furthermore, regarding the TCGA, we were limited in our ability to directly compare primary tumors with marked metastasis and those that do not metastasize. We were able to do this for TCGA pancreatic and adrenocortical cancer types finding higher activating invasion and metastasis hallmark activity for primary cancers that metastasize in comparison to those that do not. However, the abundance of nonmetastasizing samples in comparison to metastasizing samples limited the scope of the findings in most other cancer types.

To ensure comparability between datasets, each cohort of RNA-seq datasets was normalized so that the data were compared in the same format and analyzed separately. The robustness of our results was also verified by using alternative distance measures, such as those based on Spearman’s correlation and cosine similarity, that are more immune to batch effects in the TD calculation scheme.

Bearing these limitations in mind, this study asked and characterized whether the transcriptome or particular pathway of a tumor sample is closer to the tissue from which it originated or the tissue to which it metastasizes. Studying this question across cancer and data types as well as various TTs has enabled us to shed some light on this fundamental question on a large-scale manner, highlighting the importance of pathways and gene sets in certain cancer types, particularly for priming primary tumors for metastasis. More comprehensive answers to this question await the analysis of large transcriptomic datasets of paired primary tumors and their metastases, both on the bulk and single-cell levels. Nevertheless, this systematic analysis of the expression landscape of primary tumors and metastases with respect to their noncancerous OTs and TTs provides a transcriptome-wide, pan-cancer view of cancer tumors’ adaptations to their metastatic niches.

## MATERIALS AND METHODS

### Expression data collection

RNA-seq gene expression data were collected from the GEO, Xena Browser, and other sources collected into table S1A. Most were collected in fragments per kilobase of transcript per million mapped reads or count form, although some paired datasets were sourced in unique formats (e.g., upper-quartile normalized counts from phs001866) as the counts were unavailable. The gene expression data from the noncancerous sources were always converted to match the cancerous data to which they were compared; for example, GTEx count data were converted to the trimmed mean of m values–counts per million (TMM-CPM) format of the GSE184869 before comparing expression vectors. As a special case, the data for SRR2089755 were sourced from the BARRA:CuRDa browser (*42*), which contained formatted counts, instead of the original manuscript (*14*), which only contained raw FASTQ files.

### Phenotypic data collection

Phenotypic data fields of interest generally included information on whether a tumor is primary or metastatic, biopsy sites, and cancer types for all samples, as tabulated in table S1B.

### Sample filtering

The datasets were trimmed to just the relevant samples, i.e., those for which cancerous, OT, and TT gene expression data existed. The “Samples Used” columns in table S1A reflect the samples considered in the study after filtering. Each dataset had to be filtered somewhat differently as each had differently organized phenotypic data. The informative fields/columns used in filtering of the sources’ phenotype tables are given in table S1B.

### Data processing

To ensure comparability between noncancerous and cancerous datasets (table S1A), noncancerous datasets (downloaded as RNA-seq count data) were converted to the formats of the cancerous data. The counts to upper-quartile–normalized (UQ-normalized) counts conversion was done by dividing each sample’s expression by its 75th percentile value. The counts to TMM-CPM conversion was done with the “edgeR” R package (*43*). The counts matrix was fed into the “DGEList” function, after which “calcNormFactors” with parameter “method” = “TMM” and “cpm” functions were applied. In the single-cell data, all 10× counts were converted to CPM (more details in the Single-cell section below).

Then, to make the comparison between tumor samples and normal tissue simpler, median expression vectors were generated for each tissue type present in the noncancerous datasets (table S1A). For each tissue type and for each gene, independently for each dataset, the median (mean in single-cell datasets because of data sparsity) of the gene across all samples of that tissue type was taken. This generated an expression vector with the median transcriptome expression for each noncancerous tissue.

For each analysis, the list of genes was limited to those in common between all the datasets involved. These gene intersections and corresponding datasets are summarized in table S1C. All gene names were formatted as gene symbols and converted to gene symbols from ENSEMBL (ENSG) ids when necessary using the “biomaRt” package (*44*) in R using the package’s standard vignette.

Rarely in the TCGA, cancer samples were marked as having multiple TTs, in which case new “pseudo-samples” were created for a sample for each of its TT. For example, if a sample was marked as having metastases to the liver, lung, and brain, then the sample would be split into three different samples with the same gene expression as the original sample but each marked with only one TT (liver, lung, and brain, respectively).

### Sample-level TDs

For a given cancer sample, we used a TD to gauge its distance from a given normal tissue. TDs were calculated as follows: For cancer sample X and normal tissue Y, we treat the expression of the genes in X as a vector. Each vector is log_2_-transformed (if not already log-transformed from the source) and *z*-score standardized for normalization and fair comparison. TD is then the Euclidean distance between these two vectors, which provides a measure of the cancer’s similarity to the normal tissue. This is visualized in Fig. 1. Alternative measures like Spearman’s correlation and cosine-similarity–based distance were also used for computing TDs for the sake of robustness (notes S1).

### TD ratios

We used the TDs from the previous step to determine whether a cancer was closer to its tissue of origin or the tissue to which it metastasizes (TT). For a particular cancer type, a TD was taken from both the OT and the TT. The ratio of the distance to the OT over the distance to the TT was then taken. This TD ratio is a measure of which tissue the cancer is closer to. TD ratios above 1 indicate that the cancer is closer to the TT than the OT and thus is more similar to the former. TD ratios below 1, on the other hand, indicate that a cancer is closer to the OT than the TT (visualized in Fig. 1).

### Specificity analysis and specificity score

To determine whether the transcriptomic alterations in metastatic samples were specific to their TTs, we charted the landscape of TD ratios normalized by the distance between normal tissues using different TTs. Because the GTEx dataset contained 29 distinct tissues, we calculated normalized TD ratios for samples using all of these tissues as the TT in calculation, sans the OT. To account for the diversity of normal tissue gene expression profiles, which could confound such comparisons to multiple TTs, these “normalized TD ratios” were calculated by swapping out the original numerator term (TD between OT and tumor expression—a constant term in this specificity analysis) with the TD between the OT and TT. In a dataset, the normalized TD ratios generated using the actual TT of metastasis were then compared to the normalized TD ratios generated using the other TTs via one-sided Wilcoxon signed-rank tests, with FDR correction following (significance threshold of 0.1). From this, the specificity score for that dataset was defined as the number of times the normalized TD ratio distribution using the actual TT was significantly higher than the normalized TD ratio distributions using other TTs.

### Tumor purity and TD ratio residuals

Tumor purity was calculated using the “ESTIMATE” R package (*45*). For each cancer sample, the package calculated a score that is a stand-in for purity based on a single-sample GSEA. The package was then applied to every bulk dataset to obtain Spearman’s correlations between TD ratios and tumor purity—termed purity correlations (table S1D). Purity correlations were calculated for primary and metastatic samples separately in each dataset, and the resulting *P* values from the Spearman’s correlations were determined if a dataset needed tumor purity correction. Datasets that needed adjustment had their TD ratios corrected through a linear model; specifically, the residuals derived from the linear regression using the model TD ratio ~ tumor purity replaced the original TD ratios. These residuals were denoted as TD ratio residuals.

### Single-cell data processing

The single-cell dataset GSE178318 (*27*) did not come with phenotypic data, forcing us to infer the tumor and cell types of the tumor cell samples. The 10× raw counts were loaded and processed using the “Seurat” R package (*46*), and sample primary/metastatic classifications were differentiated into primary (by the substring “_CRC”), metastatic (by the substring “_LM”), and blood cells. Quality control (QC) was performed by removing samples with high mitochondrial reads (15% threshold) and only keeping samples with number of genes detected (“nFeature_RNA”) and number of molecules detected (“nCount_RNA”) within three SDs of the mean. These QC samples were then clustered using highly variable genes in principal component analysis and t-distributed stochastic neighbor embedding, after which the tumor cells (malignant/epithelial) were extracted by only keeping the two clusters that showed high *EPCAM* expression. Having the counts of just the epithelial cells (already split into primary and metastatic at this point), these were finally converted into log CPM using the “NormalizeData” function, setting “scale.factor” = “1e6.” This protocol was adapted from the Materials and Methods section of the GSE178318 publication (*27*). For the noncancerous datasets, the phenotypic information was provided as cell annotations, so only the QC step was done with the same parameters as above.

Because of the overall sparsity of the single-cell data, the number of genes used in the TD ratios analysis was limited to the 10,000 most variable genes between the noncancerous and cancer datasets. TD ratios were generated per patient for different numbers of variable genes, and 10,000 was chosen because that many genes adequately determined TD differences between primary and metastatic samples. More details are provided in the Supplementary Materials (fig. S2).

### Deconvolution analysis

To extract the epithelial/tumor cell gene expression from bulk data, we needed to first generate a signature of genes delineating each cell type. The dataset deconvolved was jnci 2018, a paired BRCA primary and brain metastatic dataset, therefore, signatures needed to be generated for BRCA primary samples and BRCA brain metastatic samples, independently. Each signature was generated by feeding annotated single-cell data (CPM format) into the CIBERSORTx (*47*) online portal using default parameters, which was then fed in conjunction with the bulk data into a high-performance computing cluster implementation of the CODEFACS algorithm (*30*) also using the default parameters. The output gene x sample matrix was filtered to just keep epithelial cells per sample keeping all genes, and it was used for downstream analysis.

For the primary BRCA data source, we derived a signature matrix of 1400 genes and nine cell types using a single-cell RNA-seq primary BRCA cohort (*48*). These nine cell types included B cells, cancer-associated fibroblasts, cancer epithelial cells (malignant), endothelial cells, myeloid cells, normal epithelial cells, plasmablasts, perivascular-like cells, and T cells. Meanwhile, for the metastatic BRCA data source, a signature matrix of 3319 genes and nine cell types using specifically the breast single-cell RNA-seq profiles from a cohort with several TTs was derived (*16*). These metastatic cell types were malignant cells, vascular smooth muscle cells, mesenchymal stem cells, metastasis-associated macrophages, endothelial cells, pericytes, astrocytes, T cells, and dendritic cells.

### Pathway selection

Hallmark Pathways were downloaded from MSigDB (*21*). These are well-defined pathways reflecting specific metabolic, signaling, or immune states. For each analysis, only pathways with sufficient overlap in the shared genes between the pathway and the dataset being analyzed were kept. In the bulk, a sufficiency threshold of 90% overlap was used, and, in the single-cell data due to sparsity, a 70% was used instead.

### Pathway-specific TD ratios

By creating a TD ratio with just the genes in a pathway rather than the entire transcriptome, we can determine whether a certain phenotypic feature of a cancer is more similar to the TT or the OT. To accomplish this, for a given pathway and tumor sample, we took the genes in that pathway and created expression vectors—one for the tumor sample and a median expression vector for the origin and target normal tissues of that tumor. Each tumor then had the TD from the OT and TT measured using only the genes for just that pathway. Just as before, the tumor sample’s TD ratio was created by dividing the TD to the OT by the TD to the TT. The pathway-specific TD ratio was calculated as the median TD ratio of samples of that cancer type. Only combinations of cancer type, primary/metastatic classification, and TT with at least three samples were considered here.

### Δ pathway-specific TD ratios

These were calculated by dividing the pathway-specific TD ratio (generated for a particular cancer type, primary/metastatic classification, and TT, in each pathway) by the median of the TD ratios generated with the same cancer type, primary/metastatic classification, and TT, but using all genes (referred to as the all-genes TD ratio). Thus, these Δ pathway–specific TD ratios represented if a pathway was closer to an OT/TT compared to the overall transcriptome’s tendency for a given set of samples. This process is summarized and visualized in fig. S6.

### Δ pathway-specific PMT scores

The process for calculating these Δ pathway–specific PMT scores is similar to the process for calculating the Δ pathway–specific TD ratios. We simply substituted the TD ratio calculations for PMT score calculations. PMT score for any given metastatic sample is defined as ED(metastatic sample expression, paired primary sample expression) / ED(metastatic sample expression, TT), “ED” being Euclidean distance. The details are provided below.

PMT scores were used to compare a metastatic sample’s similarity to its TT and paired primary tumor. PMT scores above 1 indicate more closeness in expression to the TT, and vice versa for closeness to the paired primary tumor. These were made “pathway-specific” by calculating the Euclidean distance in the above calculation with only the genes in that pathway. The pathway-specific PMT score was then the median score of all samples in a certain cancer type and with a certain TT (e.g., BRCA brain metastases), for any given pathway. Last, the Δ pathway–specific PMT scores were calculated by dividing the pathway-specific PMT score by the median of the PMT scores generated with the same cancer type and TT group (e.g. BRCA brain metastases) but using all genes. Thus, these Δ pathway–specific PMT scores represented if a pathway was closer to an OT/TT compared to the overall transcriptome’s tendency for a given set of samples.

### Empirical *P* value generation and correction

Empirical *P* values were calculated for each pathway as follows: For 10,000 iterations, a random set of genes the same size as the pathway was selected. The Δ pathway–specific TD ratios were then calculated for these random sets, whereafter the empirical *P* value (*P*) was calculated as such: Let *r* be the number of times the random set of genes’ ratios were larger than or equal to the ratio for the specific pathway and let *n* be the number of random gene sets (10,000 here).

Then, *P* = (*r* + 1)/(*n* + 1), in accordance with standard empirical *P* value generation protocol (*49*). *P* values were adjusted by setting them to minimum of *P* and 1 – *P* as a Δ pathway–specific TD ratio below (more similar to OT) or above (more similar to TT) those from the random gene sets could be significant and FDR (multiple hypothesis) corrected (*50*). In this way, the empirical *P* values represented how significant the shift in expression was for any pathway in a cancer type from the OT to the TT, or vice versa.

### Acquisition of the cancer hallmark gene sets

The 10 main cancer hallmarks, originally proposed by Hanahan and Weinberg in 2011, were mapped to gene sets by Iorio *et al.* (*40*) in 2018, consisting of a total of 374 orthogonal pathway gene sets (*22*). The gene sets were all taken directly from that publication and used in this work.

### GSEA on cancer hallmarks

GSEA (*39*) was performed on paired datasets to determine whether the hallmarks were more enriched/active in primary or metastatic samples. For any given paired dataset, (i) fold changes were calculated for all shared genes between the primary and metastatic (metastatic divided by primary) samples using the unlogged/not log-transformed gene expressions. Specifically, the fold change for any given gene x (within a dataset) was defined as log_2_(geometric mean of expression of gene x in metastatic samples/geometric mean of expression of gene x in primary samples). Then, (ii) for each hallmark gene set, to account for the rare instance of distinct genes in the sets and not in the cancer expression, we removed genes not in the cancers’ expression datasets. Last, after setting the random seed for consistency, (iii) we fed the pruned pathways and fold changes into the “fgseaMultiLevel” function from the ‘fgsea’ R package (*51*) using 10,000 iterations (50,000 for rare instances where 10,000 resulted in blank output and a suggestion to do more iterations). Default parameters were used for fgseaMultiLevel otherwise. The NESs and FDR-corrected *P* values were taken from the output of the fgseaMultiLevel function for downstream analysis.

Sampling of the GSEA results for random gene sets was also performed using the same three steps as the previous paragraph but for 10,000 randomly sampled gene sets from the cancer sample expression matrix genes—the enrichment results of which are shown in fig. S9A and described in notes S2. Furthermore, to run the GSEA on the individual pathways that composed certain hallmarks, those pathways (also gene sets) were simply substituted in for the hallmarks and ran through the same three steps as above.

### GSVA on cancer hallmarks

To determine the hallmarks’ activities in the unpaired setting, GSVA (*41*) was used to compare primary and metastatic samples. The unpaired data, consisting of one large normalized and scaled gene expression matrix, was fed along with the hallmark gene sets into the “gsva” function from the gsva R package, setting method = gsva. The activity score outputs per sample were then compared via two-sided Wilcoxon rank-sum tests. To check whether the sampling scores were biased toward higher values in primary or metastatic samples in general, we calculated scores with random gene sets, as done in the GSEA (notes S2).

### Statistical analysis

To compare the distributions of TD ratios in primary and metastatic samples, one-sided Wilcoxon signed-rank tests (for paired data) and one-sided Wilcoxon rank-sum test (for unpaired data) were used (*52*). When applicable, multiple hypothesis correction (FDR) using the Benjamini and Hochberg method was done to correct *P* values (*50*). Empirical *P* value generation for the pathways analysis is described in a previous paragraph. The distributions of cancer hallmark activities as calculated by the gene-set variation analysis were compared via two-sided Wilcoxon rank-sum tests. All statistical analyses were performed in R.

## Supplementary Materials

### The PDF file includes:

Legends for tables S1 to S4 Supplementary Text (Notes S1 to S3) Figs. S1 to S9
